# Inactivation of the PHD3-FOXO3 axis blunts the type I interferon response in microglia and ameliorates Alzheimer’s disease progression

**DOI:** 10.1126/sciadv.adu2244

**Published:** 2025-05-28

**Authors:** Manuel A. Sanchez-Garcia, Nieves Lara-Ureña, Rosana March-Diaz, Clara Ortega-de San Luis, Silvia Quiñones-Cañete, Bella Mora-Romero, Juan M. Barba-Reyes, Daniel Cabello-Rivera, Carmen Romero-Molina, Antonio Heras-Garvin, Victoria Navarro, Jose Lopez-Barneo, Marisa Vizuete, Javier Vitorica, Ana M. Muñoz-Cabello, Ana B. Muñoz-Manchado, Matthew E. Cokman, Alicia E. Rosales-Nieves, Alberto Pascual

**Affiliations:** ^1^ Instituto de Biomedicina de Sevilla (IBiS), Hospital Universitario Virgen del Rocio/CSIC/Universidad de Sevilla, 41013 Seville, Spain.; ^2^ Centro de Investigacion Biomedica en Red sobre Enfermedades Neurodegenerativas (CIBERNED), Madrid, Spain.; ^3^ Departamento de Biología Celular, Facultad de Biología, Universidad de Sevilla, Seville, Spain.; ^4^ Departamento de Neurociencias. Unidad de Biología Celular. Instituto de Investigación e Innovación Biomédica de Cádiz (INiBICA). Universidad de Cádiz, Cadiz, Spain.; ^5^ Departamento de Fisiología Médica y Biofísica, Universidad de Sevilla, Seville, Spain.; ^6^ Departamento de Bioquímica y Biología Molecular, Facultad de Farmacia, Universidad de Sevilla, Seville, Spain.; ^7^ Laboratory of Molecular Neurobiology, Department of Medical Biochemistry and Biophysics, Karolinska Institutet, Stockholm SE-17177, Sweden.; ^8^ Ciber of Mental Health (CIBERSAM), ISCIII, 28029 Madrid, Spain.; ^9^ Hypoxia Biology Laboratory, Francis Crick Institute, London NW1 1AT, UK.

## Abstract

Microglia respond to Alzheimer’s disease (AD) with varied transcriptional responses. We show that oligomeric Aß (oAß) induces the expression of *Hif1a* and *Egln3* in microglia in vitro, together with the transcription of the type I interferon signature (IFNS) genes in a PHD3-dependent manner. We identify FOXO3 as a repressor of IFNS, whose abundance decreases upon PHD3 induction in response to oAß. In vivo, loss of PHD3 correlates with abrogation of the IFNS and activation of the disease-associated microglia signature, an increase in microglia proximity to Aß plaques and phagocytosis of both Aß and small plaques. PHD3 deficiency mitigated the Aß plaque–associated neuropathology and rescued behavioral deficits of an AD mouse model. Last, we demonstrate that microglial PHD3 overexpression in the absence of Aß pathology is sufficient to induce the IFNS and behavioral alterations. Together, our data strongly indicate that the inactivation of the PHD3-FOXO3 axis controls the microglial IFNS in a cell autonomous manner, improving AD outcome.

## Teaser

PHD3 inhibition slows down the pathology and behavioral decline of an Alzheimer’s disease mouse model.

## INTRODUCTION

Microglia are brain-resident macrophages that play relevant roles in brain homeostasis, including myelin maintenance, efferocytosis of defective neurons, and vascular support (*1*, *2*). Microglia are also involved in neurodegenerative disorders, contributing to the brain responses to local damage (*2*). Analysis of genetic risk factors in Alzheimer’s disease (AD) has placed microglia in the spotlight of intense research by revealing that polymorphisms in genes with prominent or exclusive functions in microglia are linked to AD (*3*). These polymorphisms are implicated in varied functional modules like lipid transport, endolysosomal functioning, and microglial migration and phagocytosis, among others (*3*). In AD, microglia cluster (*4*) and establish a protective barrier around senile plaques (*5*–*8*), although they may also contribute to the progression of the disease by activating a pro-inflammatory phenotype (*9*–*12*). As innate immune cells, microglia express a variety of cell-surface receptors (*13*), which may bind to soluble Aβ peptide oligomers and fibrils (*11*) or to other molecules released from injured cells. Aß activates diverse signaling pathways, including a common microglial neurodegenerative phenotype observed in disease-associated microglia signature (DAM) (*14*–*17*) and, among others, interferon (IFN) (*18*–*22*) and hypoxia-inducible factor 1 (HIF1)–dependent signatures (*23*). However, how these transcriptional signatures are regulated, how they interact with each other, and which are the functional consequences of their activation are only starting to be deciphered.

We have previously shown that loss of endothelial cells around Aß plaques (*24*) induces a HIF1-mediated transcriptional program in Aß plaque–associated microglia (AßAM) (*23*) and that HIF1 activity under hypoxia limits the microglial defensive potential against the disease (*23*). HIF1 is transcriptionally regulated by different signaling pathways, including the mechanistic/mammalian target of rapamycin pathway (*25*), which has been involved in the activation of AßAM (*23*, *26*). HIF1 protein stability is also modulated in an oxygen-dependent manner. In the presence of oxygen, HIF prolyl hydroxylases (PHD1 to PHD3) hydroxylate the HIF1α subunit in specific prolines triggering its ubiquitination by von Hippel–Lindau (VHL) and subsequent proteasomal degradation (*27*). Under low oxygen levels, HIF1α accumulates, translocates to the nucleus, and activates a complex cell-type–dependent transcriptional program (*27*). In monocyte-derived cells, HIF1 promotes the transcription of the pro-inflammatory cytokines the tumor necrosis factor–α (TNFα) and interleukin-1ß (IL-1ß) (*28*, *29*) and induces a switch from oxidation to fermentation of glucose (*28*), a metabolic adaptation that has also been proposed in AßAM dysfunction (*30*, *31*). A recent article has postulated HIF1, together with Forkhead box O3 (FOXO3) and Forkhead box protein P2 (FOXP2), as a main player in the transition between low to high inflammatory profile in human induced pluripotent stem cell (iPSC)–derived microglia (*32*). However, we lack in vivo data of the function of these transcription factors in AD. *Egln3*, encoding for PHD3, is a HIF1-target gene with known functions in the regulation of innate immunity (*33*–*37*). This gene has previously been found as one of the most unregulated transcripts in AßAM (*23*) and interacts with FOXO3 (*38*), and its homolog PHD1 regulates the stability of FOXO3 (*39*). This work aims to characterize the contribution of HIF1, PHD3, and FOXO3 to the functional and transcriptional responses of AßAM and their involvement in Alzheimer’s progression.

## RESULTS

### HIF1 activates *Egln3* transcription in AßAM without altering cytokine production

**Fig. 1.**
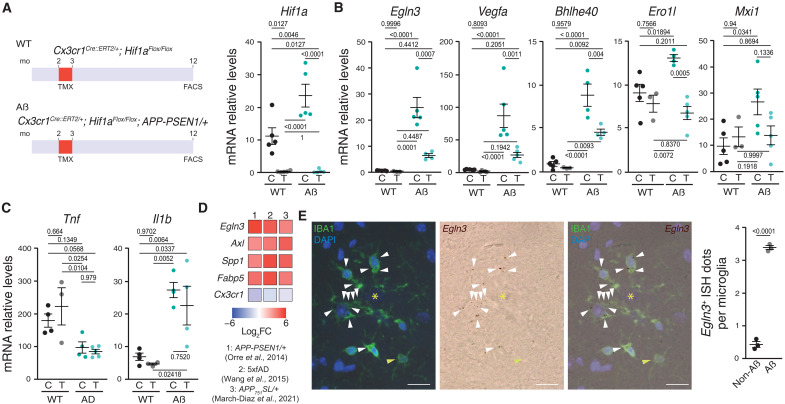
AßAM activate the expression of *Egln3* through HIF1. (**A** to **C**) Left: Mouse models used. mo, -month-old; TMX, tamoxifen. Right: *Hif1a* (A) and HMM-included genes (B and C) mRNA expression in FACS-isolated microglia from 12-month-old *Cx3cr1^CRE::ERT2/+^*; *Hif1a^Flox/Flox^* control (C)– and tamoxifen (T)–treated wild-type (WT) and *APP-PSEN1/+* (Aß) mice were analyzed by qRT-PCR. (**D**) Heatmap of the log_2_ fold change (FC) of *Egln3*, *Axl*, *Spp1*, *Fabp5*, and *Cx3cr1* genes in three different Aß-depositing models (*16*, *23*, *43*). (**E**) In situ hybridization (ISH) of *Egln3* mRNA, immunohistochemistry for microglia (IBA1), and nuclear [4′,6-diamidino-2-phenylindole (DAPI)] staining in brain sections of 12-month-old *APP-PSEN1/+* mice proximal (white arrowheads) and distal (yellow arrowheads) to Aβ plaques (yellow asterisk). Scale bars, 10 μm. Right: Quantification of the number of Egln3^+^ dots per microglia (IBA^+^) distal (non-Aß) and proximal (Aß) to Aß plaques. All data are presented as means ± SEM. Individual points in the graphs indicate independent mice used per experiment. *P* values from ANOVA with Tukey’s posttest (C) or two-tailed Student’s *t* test (E).

We have previously shown that AßAM express the HIF1/hypoxia-induced module (HMM) and that overactivation of HIF1 leads to microglial quiescence (*23*). However, the role of HIF1 in homeostatic or activated microglia has not been addressed. In monocyte-derived cells, HIF1 is associated with the transcription of the pro-inflammatory cytokines IL-1ß and TNFα (*28*, *29*) and a similar role has been suggested in microglia (*30*, *31*, *40*). To test the role of HIF1 in microglia, we generated a HIF1α-deficient mouse model with and without Aß deposition (*Cx3cr1^Cre::ERT2/+^*; *Hif1a^Flox/Flox^* ± *APP-PSEN1/+*). We treated 2-month-old mice with tamoxifen in the diet for 30 days, aged the mice to further 10 months, and isolated the microglia by fluorescence-activated cell sorting (FACS). We confirmed a strong down-regulation of *Hif1a* mRNA levels in both wild-type (WT) and Aß-depositing mouse models in tamoxifen-treated *Cx3cr1^Cre::ERT2/+^*; *Hif1a^Flox/Flox^* mice (Fig. 1A), a finding in line with previous observations in *Cx3cr1^Cre::ERT2/+^*; *Hif1a^Flox/Flox^* mouse primary microglial cultures (*23*). Accordingly, *Hif1a* itself and genes belonging to the HMM were induced in AßAM and down-regulated in HIF1-deficient microglia, including *Egln3*, *Vegfa*, *Bhlhe40*, *Ero1l*, and *Mxi1* (Fig. 1B). Unexpectedly, *Tnf* was not induced by the pathology nor regulated by HIF1, and *Il1b* was induced in AßAM but not regulated by HIF1 (Fig. 1C), strongly suggesting differential consequences of HIF1 activation in microglia to monocyte-derived cells.

To further investigate the role of HIF1 in AßAM, we focus on *Egln3*, a well-known HIF1-target gene (*41*) that is involved in the modulation of HIF1 and FOXO3 (*38*, *39*, *42*). In the immune system, PHD3 has a major relevance in the regulation of inflammation in several cell types including macrophages (*33*–*37*), suggesting its implication on HIF1/FOXO3-mediated control of innate immunity.

To further characterize the expression of PDH3 in microglia, we reanalyzed the expression of *Egln3* mRNA in microglia isolated by FACS from several Aß-depositing models (*16*, *23*, *43*) and compared with the expression changes in some genes highly expressed by AßAM (Fig. 1D). This approach enabled us to observe a substantial induction of *Egln3* across different mouse models. To confirm the transcriptomic data, we performed in situ hybridization combined with immunofluorescence. *Egln3* mRNA colocalized with Ionized calcium-binding adaptor molecule 1 (IBA1)–immunoreactive cells in the proximity of Aß deposits but showed low expression in distal-to-plaque homeostatic microglia (Fig. 1E). Together, our data indicate that HIF1 contributes to the expression of PDH3 in AßAM.

### PHD3 regulates FOXO3, a repressor of the IFNS in microglia

**Fig. 2.**
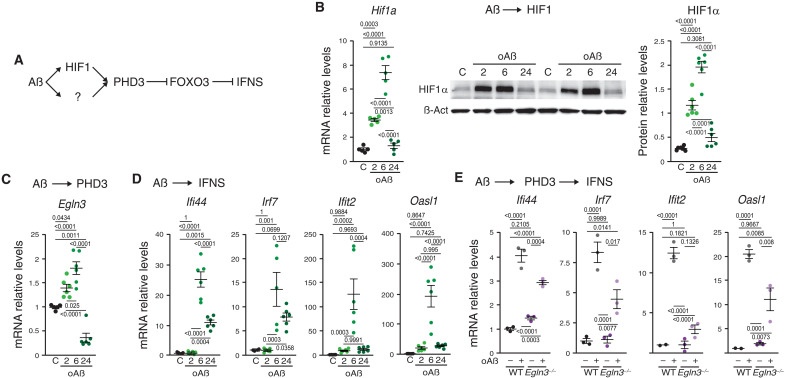
PHD3 regulates the microglial IFNS in response to Aß. (**A**) Hypothetical pathway involved in the activation of IFNS by oAß. Arrows indicate activation, and cross-lines indicate repression. (**B** to **D**) *Hif1a* (B), *Egln3* (C), and IFNS (D) mRNAs; and HIF1α protein (B) levels in primary neonatal microglial cell cultures exposed to control (C) or oligomeric Aß (oAß) for 2, 6, or 24 hours. *P* values from analysis of variance (ANOVA) with Tukey’s posttest. (**E**) mRNA levels of antiviral genes were estimated in primary neonatal microglial cell cultures from WT or *Egln3^−/−^*mice exposed to control (−) or oAß (+) for 24 hours. *P* values from ANOVA with Tukey’s posttest. All data are presented as means ± SEM. Individual points in the graphs indicate independent mice used per experiment.

**Fig. 3.**
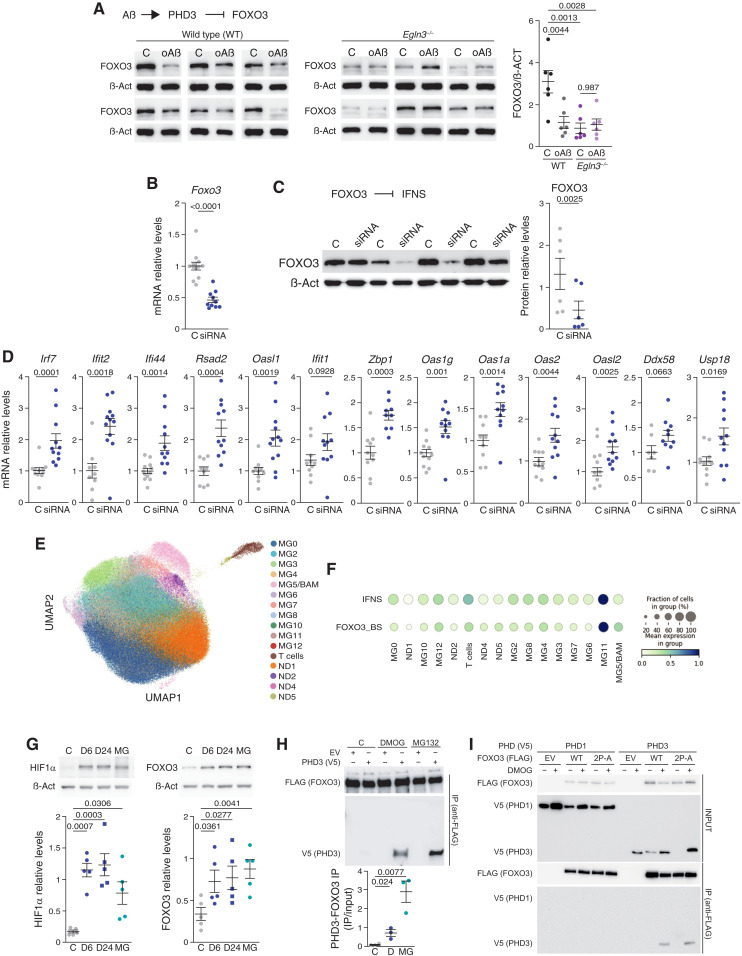
PHD3 modulates IFNS through FOXO3. (**A**) FOXO3 protein levels in microglial cultures: WT and *Egln3^−/−^* mice exposed to control (C) or oAß. *P* values: ANOVA and Tukey’s posttest. (**B** and **C**) *Foxo3* mRNA (B) and FOXO3 protein (C) levels (C or anti-*Foxo3* siRNA). *P* values: Mann-Whitney’s test (B) or two-tailed Student’s *t* test (C). (**D**) IFNS mRNA levels in microglial cultures as in (B) and (C). *P* values: Mann-Whitney’s test for *Irf7*, *Ifit2*, *Oas1g*, and *Ddx58*; and two-tailed Student’s *t* test for *Ifi44*, *Rsad2*, *Oasl1*, *Ifit1*, *Zbp1*, *Oas1a*, *Oas2*, *Oasl2*, and *Usp18*. (**E**) UMAP plot illustrating 11 microglial states (*32*). (**F**) Dot plot with expression of IFN and FOXO3 binding site (BS) gene signatures. Each circle represents mean expression level (color intensity) and proportion of cells expressing IFN or FOXO3_BS signatures (dot size). MG11 displays significant overexpression of both IFN (log fold change, 1.7; *P* value < 0.05) and FOXO3_BS (log fold change, 2.03; *P* value < 0.05) signatures. (**G**) HIF1α (left) and FOXO3 (right) protein levels in microglial cultures (C, DMOG (D; 6 or 24 hours), or MG132 [MG; 24 hours)]. *P* values: ANOVA and Tukey’s posttest. (**H**) HEK293T cells transfected: FOXO3-FLAG and empty (EV) or PHD3-V5 vector; incubated with vehicle (C), DMOG (D), or MG132 (M) for 24 hours. Top: Western blots: anti-FLAG (FOXO3) and anti-V5 (PHD3) after immunoprecipitation (IP) with anti-FLAG. Bottom: Quantification of top panels. *P* values: Student’s *t* test. (**I**) HEK293T cells transfected: PHD1-V5 or PHD3-V5 and empty (EV), WT FOXO3-FLAG, or P_426_A/P_437_A mutant (2P-A) FOXO3-FLAG vector; and with (+) or without (−) DMOG for 24 hours. Top: Western blots: Anti-V5 (PHD1 and PHD3) and anti-FLAG (FOXO3) in proteins extracts (INPUT) Bottom: After immunoprecipitation (IP) with anti-FLAG. All data presented are means ± SEM. Individual points represent the *n* and are independent microglial (A to D and G) or HEK293T (H and I) cultures.

As described before, PHD3 has a role in the innate immune system (*33*–*37*), is strongly up-regulated in AßAM, and interacts with FOXO3 (*38*), a protein that could be involved in the transition from low to high inflammatory microglia (*32*). Mechanistically, FOXO3 represses the transcription of antiviral IFN target genes in macrophages through direct binding to their promoters (*44*). IFN-expressing microglia have recently been identified in models of AD and in controls and AD human brains (*18*–*22*). The activity of IFN stimulated genes in microglia has been associated with decreased synaptic markers and worsening of behavioral function in AD mouse models (*20*, *45*) and with physiologic pruning of synapsis during development (*46*). We therefore hypothesized that a HIF1-PHD3-FOXO3 axis may be controlling the IFN signature (IFNS) expression in microglia (Fig. 2A). To understand the role of PHD3 induction in microglia, we exposed primary neonatal microglial cell cultures to oligomeric Aß (oAß), which activates microglia in contrast to monomeric Aß treatment (*47*). HIF1α was strongly increased both at mRNA and protein levels (Fig. 2B), paralleled by its target gene *Egln3* (Fig. 2C and fig. S1A). Then, we verified that oAß, but not Aß, treatment induced in vitro the expression of the IFNS, including the master regulator *Irf7* (Fig. 2D and fig. S1A) (*48*), similar to the induction of the type I IFN response observed in FACS-isolated AßAM from *5xfAD/+* and *APP_751_SL/+* mouse models (fig. S1B and tables S1 to S3). The up-regulation of *Hif1a*/*Egln3* precedes that of the IFNS, suggesting a potential involvement in its regulation. To evaluate the role of PHD3 in the up-regulation of the IFNS by oAß, we performed primary neonatal cultures of control and PHD3-deficient microglia (*Egln3^−/−^* mice) and observed a significant down-regulation of the IFNS in the absence of PHD3 (Fig. 2E). However, the IFNS expression was not fully abolished by PHD3 deficiency after oAß treatment, suggesting that other pathways, like the cyclic guanosine 5′-monophosphate–adenosine 5′-monophosphate synthase–stimulator of IFN gene pathway, described as regulators of this signature in microglia (*49*), could also be involved in the IFNS induction by oAß in vitro. oAß treatment reduced FOXO3 protein levels in WT but not in *Egln3^−/−^* (Fig. 3A) microglia, strongly indicating that PHD3 controls FOXO3 abundance. Notably, *Egln3^−/−^* microglia presented reduced levels of FOXO3 in the absence of treatment, suggesting that, in the absence of PHD3, cells required less FOXO3 (Fig. 3A). To formally demonstrate that the levels of FOXO3 are directly linked with the expression of the IFNS, we knockdown FOXO3 expression in primary neonatal microglial cell cultures. The reduction in FOXO3 was verified at the mRNA and protein levels (Fig. 3, B and C) and resulted in the activation of the IFNS (Fig. 3D), suggesting that, like in peripheral macrophages (*44*), FOXO3 represses IFNS transcription in microglia.

To test the role of FOXO3 in regulating the transcription of the IFNS in microglia in vivo, we first reanalyzed the transcriptomic data from acutely isolated AßAM from an AD mouse model and observed a prominent enrichment of the genes containing experimentally demonstrated FOXO3 DNA binding sites (BSs; fig. S1C) (*44*). Reanalysis of single-nucleus RNA sequencing (snRNA-seq) data from a previous study (*32*) was used to validate the control of the IFN-expressing microglia by FOXO3 in humans. We clustered the cells using the same markers than in (*32*) (Fig. 3E) and clearly identified the IFNS-expressing microglia (Fig. 3F; MG11). We then interrogated the expression of the genes controlled by FOXO3 in the different microglial types, observing a clear contribution of FOXO3-regulated genes to human IFNS-expressing microglia (Fig. 3F and table S2).

PHDs may regulate FOXO3 stability through hydroxylation of FOXO3 itself or other FOXO3 regulators or by an alternative mechanism involving direct interaction (*38*, *39*); therefore, we evaluated whether PHD3 activity could be defining the levels of FOXO3 in microglia. To this end, we first exposed primary cultures of neonatal microglia to the pan-PHDs inhibitor dimethyloxalylglycine (DMOG) and evaluated the levels of HIF1α, as a control, and FOXO3. FOXO3 was accumulated with a similar kinetic to HIF1α (Fig. 3G), suggesting that PHDs control the levels of FOXO3 in microglia. Because FOXO3 protein levels are regulated by proteasomal degradation (*39*), MG132 proteasome inhibitor was used to identify accumulation of HIF1α and FOXO3 in microglia (Fig. 3G). Last, to evaluate whether a physical interaction exists between PHD3 and FOXO3 and its dependence on PHDs hydroxylase activity, we overexpressed both tagged-proteins in human embryonic kidney (HEK) 293T cells and performed co-immunoprecipitation studies including PHD1 as a control. First, we validated the technique by using HIF1α and observed co-immunoprecipitation between both PHD1 and PHD3 and HIF1α after PHDs inhibition with DMOG (fig. S1D), a phenomenon previously described, as DMOG increases the interaction time between the enzyme and its substrate due to the slowdown of the catalytic cycle [substrate trapping (*38*)]. FOXO3 co-immunoprecipitated with PHD3 and not with PHD1 in the presence of DMOG (Fig. 3, H and I, and fig. S1E) or MG132 (Fig. 3H and fig. S1E; only PHD3), suggesting a specific interaction between both proteins. FOXO3 direct hydroxylation has not been reproduced in vitro (*50*), but we observed a clear interaction with FOXO3 by inhibiting PHDs activity. To test whether the interaction was dependent on direct hydroxylation of prolines in FOXO3, we mutated the two prolines described as hydroxylated in FOXO3 (*39*) and observed the same interaction in the presence but not in the absence of DMOG (Fig. 3I), strongly suggesting that DMOG may be inhibiting the activity of PHDs over other targets or, alternatively, induces conformational changes in PHD3 that stabilizes the interaction with FOXO3. Together, these results indicate that oAß induces HIF1 and PHD3, which reduces the stability of FOXO3 in primary microglial cultures leading to the up-regulation of the IFNS.

### PHD3 deficiency abolishes the IFN response in AßAM

**Fig. 4.**
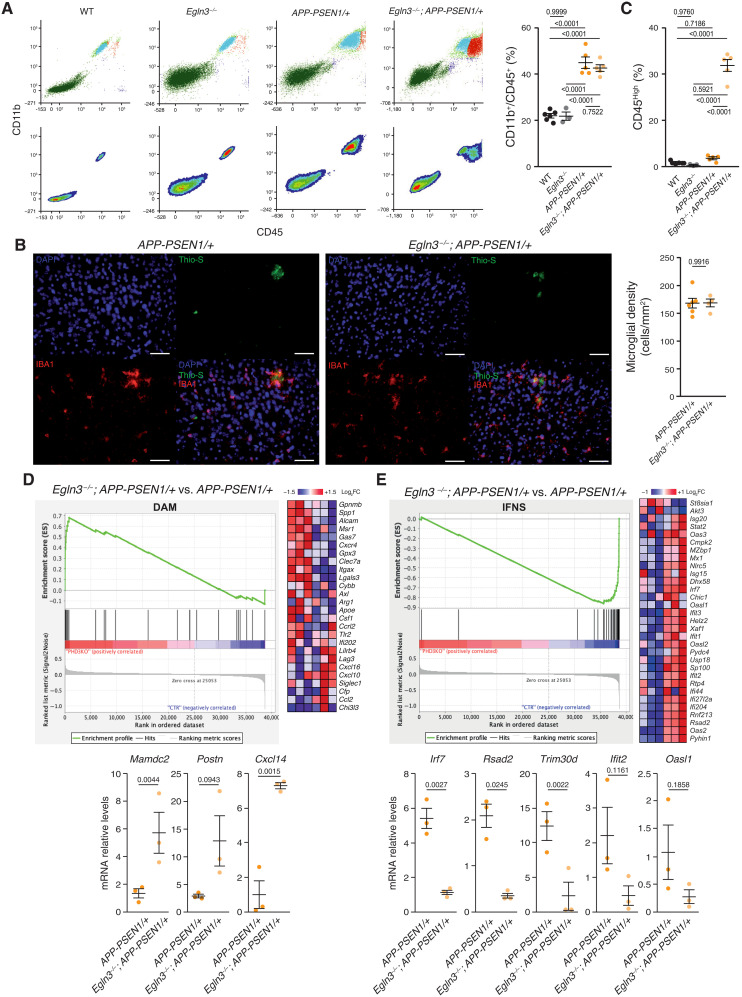
PHD3 regulates the IFNS in AßAM from an AD mouse model. (**A**) Adult microglia from 12-month-old WT, *Egln3^−/−^*, *APP-PSEN1/+*, and *Egln3^−/−^*; *APP-PSEN1/+* mice were characterized by surface expression of CD45 and CD11b. Top: Each dot represents one fluorescent event. Bottom: The density plots from the data in the top panels. Right: Quantification of the percentage of CD45/CD11b positive events. *P* values from ANOVA with Tukey’s posttest. (**B**) Stereological estimation of microglial density. Left: Representative cortical coronal sections from *APP-PSEN1/+* and *Egln3^−/−^*; *APP-PSEN1/+* mice immunostained with an anti-IBA1 antibody and stained with thioflavin-S (Thio-S). DAPI was used to label nuclei. Scale bars, 50 μm. Right: IBA1-positive cells density in the cortex of *APP-PSEN1/+* and *Egln3^−/−^*; *APP-PSEN1/+* mice. *P* values from two-tailed Student’s *t* test. (**C**) Quantification of the percentage of CD45^High^ positive events in (A). Low and high populations were determined using the density plots shown in (A, bottom). *P* values from ANOVA with Tukey’s posttest. (**D** and **E**) Top: GSEA of FACS-isolated microglia from 12-month-old *Egln3^−/−^*; *APP-PSEN1/+* versus *APP-PSEN1/+* showing DAM (D) and IFNS (E) enrichment plots (left) and the heatmaps (right) of the top 30 ranking leading edge genes. FC, fold change. Bottom: qRT-PCR analysis of the expression of several genes induced (left) or repressed (right) in *Egln3^−/−^*; *APP-PSEN1/+* microglia. *P* values from Student’s *t* test. All data are presented as means ± SEM. Individual points in the graphs indicate independent mice used per experiment.

PHD3 has low expression in the brain (*51*) and PHD3 inactivation has a minor systemic phenotype (*52*); therefore, and to further understand the role of PHD3 in AßAM, we generated a mouse model combining lack of PHD3 with Aß deposition (*Egln3^−/−^*; *APP-PSEN1/+*). First, we compared the percentage of CD11b^+^/CD45^+^ microglia from 12-month-old WT, *Egln3^−/−^*, *APP-PSEN1/+*, and *Egln3^−/−^*; *APP-PSEN1/+* mice using flow cytometry (fig. S2A). No differences were observed between WT and *Egln3^−/−^* mice in the absence of Aß accumulation (Fig. 4A), in agreement with the low levels of *Elgn3* reported in nonreactive microglia (Fig. 1, D and E). By contrast, the deposition of Aß in *Egln3^−/−^*; *APP-PSEN1/+* and *APP-PSEN1/+* mice induced a similar increase in the percentage of CD11b^+^/CD45^+^ microglial cells (Fig. 4A). This finding was further confirmed by stereology-assisted counting of IBA1-immunoreactive microglia in the cortex of control and PHD3-decificient Aß-depositing models (Fig. 4B). However, although the total number of microglial cells did not differ between *Egln3^−/−^*; *APP-PSEN1/+* and *APP-PSEN1/+* mice, microglial cells expressed higher levels of CD45 in AD mice lacking PHD3 (Fig. 4C), suggesting a change in activation status (*53*).

Second, to characterize the activation state of PHD3-deficient microglia in an AD mouse model, we isolated microglia using FACS and performed global transcriptional expression profiling (table S4). As expected, *Egln3* was the most down-regulated gene (table S4), and gene set enrichment analysis (GSEA) showed up-regulation of the DAM signature (Fig. 4D and table S5). Correspondingly, genes strongly up-regulated in AD mouse models (*23*), including *Mamdc2*, *Postn*, and *Cxcl14*, were further increased by PHD3 deficiency (Fig. 4D), suggesting a different microglial activation.

To further understand the changes taking place in the microglia of PHD3-deficient AD mice, we focused on down-regulated pathways. Microglia is characterized by a strong induction of several transcriptomic modules when responding to external or internal stimuli like neurodegeneration (*23*, *54*). However, transcriptional down-regulation is weakly associated with activation of microglia, with the exception of a few homeostatic genes (*14*, *15*, *17*). In agreement with our in vitro results, the most prominent gene sets down-regulated in *Egln3^−/−^*; *APP-PSEN1/+* mice included the Nod-like, Toll-like, and Rig-I–like receptor signaling pathways and the cytosolic DNA sensing pathway (fig. S2B and table S6). These pathways are related to antiviral responses mediated by IFN, which are up-regulated in AßAM. To statistically evaluate the relevance of the IFNS in PHD3-deficient AD microglia, we ran GSEA, observing a strong down-regulation of the IFNS in AD mouse models lacking PHD3 (Fig. 4E and table S7). Last, we detected a negative enrichment in the genes containing experimentally demonstrated FOXO3 DNA BSs (fig. S2C and table S7) (*44*), reinforcing the idea that FOXO3 represses IFNS via PHD3. We confirmed our microarray data using quantitative reverse transcription polymerase chain reaction (qRT-PCR; Fig. 4E) for a set of genes including *Irf7*, a key player in the establishment of the antiviral response (*48*). Together, our data strongly suggest that PHD3 contributes to the induction of the IFNS in AßAM.

### PHD3 deficiency improves functional microglial responses to Aß in vivo

**Fig. 5.**
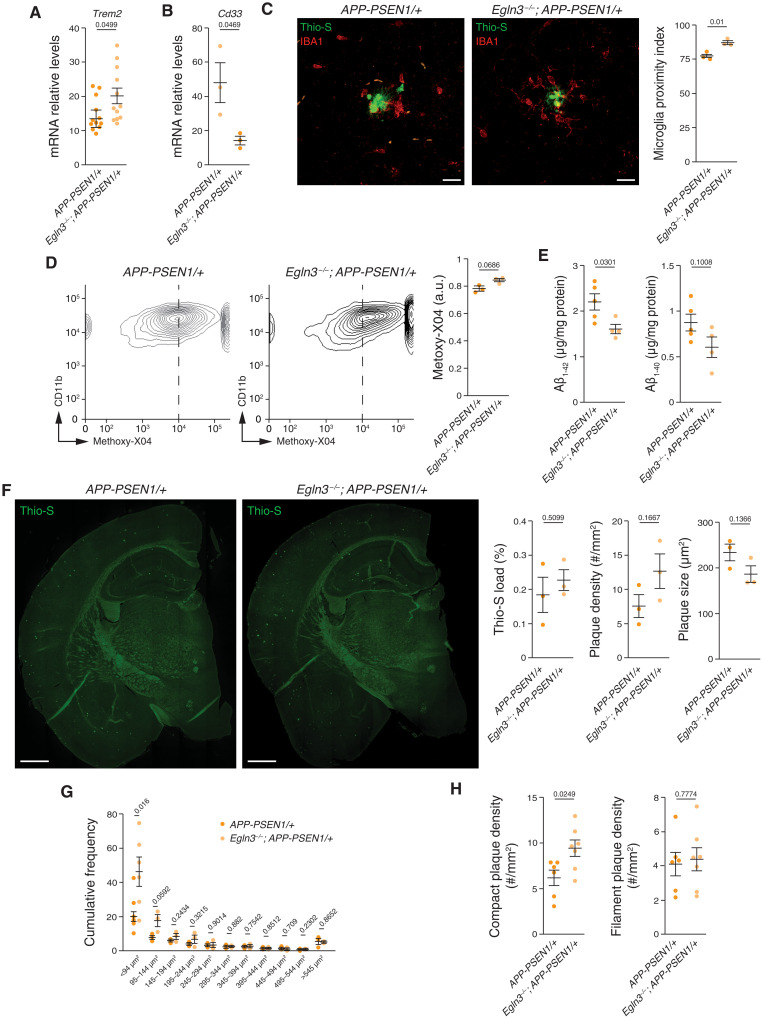
PHD3 limits microglial proximity to Aß plaques. (**A** and **B**) *Trem2* and *Cd33* mRNA levels in adult microglia isolated from *APP-PSEN1/+* and *Egln3^−/−^*; *APP-PSEN1/+* mice using qRT-PCR. (**C**) Left: Representative cortical coronal sections from *APP-PSEN1/+* and *Egln3^−/−^*; *APP-PSEN1/+* mice immunostained with an anti-IBA1 antibody and stained with Thio-S. Scale bars, 20 μm. Right: Microglia proximity index: number of IBA1-positive cells in contact with Aß plaques per total microglia within a 40-μm halo around the plaques. (**D**) Quantification of Aβ phagocytosis by flow cytometry (left and center) of microglia isolated from *APP-PSEN1/+* and *Egln3^−/−^*; *APP-PSEN1/+* mice 3 hours after intraperitoneal injection of methoxy-X04. Right: Relative measurements. *P* values from two-tailed Student’s *t* test. (**E**) Quantification of Aβ_1-42_ and Aβ_1-40_ of half cortical extracts from 6-month-old *APP-PSEN1/+* and *Egln3^−/−^*; *APP-PSEN1/+* mice by enzyme-linked immunosorbent assay (ELISA). a.u., arbitrary units. (**F** to **H**) Representative cortical coronal sections from *APP-PSEN1/+* and *Egln3^−/−^*; *APP-PSEN1/+* mice stained with Thio-S and plaque analysis. Scale bars, 500 μm. Right: Thio-S load, plaque density, and plaque size (F); cumulative frequency of Thio-S plaques (G); compact and filamentous plaque density (#, number) (H). All data are presented as means ± SEM. Individual points in the graphs indicate independent mice used per experiment. *P* values from two-tailed Student’s *t* test.

With the exception of the genes involved in the IFNS, most of genes regulated in the absence of PHD3 were genes already induced in AßAM, suggesting that PHD3 mutation increases the microglial responses to Aß. We speculate that the reduction of the antiviral module by PHD3 deficiency enables the up-regulation of other functional modules, like DAM. To investigate the functional consequences of PHD3 deficiency in AßAM, we first evaluated two genes involved in the capacity of microglia to respond to Aß accumulation and linked genetically to AD, *Trem2* (*55*–*57*) and *Cd33* (*58*). A loss-of-function mutation in *Trem2* (R47H) is one of the strongest risk factors for AD and TREM2 deficiency associates with a worsening of AD models [for a review, see (*59*)]. PHD3 limits *Trem2* expression in AD microglia, as its absence produces a further induction of the gene (Fig. 5A). Conversely, PHD3 absence correlated with a decrease in the *Cd33* expression in microglia, whose elevated abundance in AD brains is known to limit microglial phagocytic capacity (Fig. 5B) (*58*). Full activation of the DAM signature depends on TREM2 activity (*15*), a signature that contains genes related to the ability of microglia to cluster around and phagocyte Aß plaques (*15*, *43*), increasing the defensive capacity of microglia (*8*). Therefore, we tested whether the microglial spatial disposition could be altered by PHD3 deficiency by analyzing their proximity to Aß plaques. We observed a significant increase in the microglial proximity index in *Egln3^−/−^*; *APP-PSEN1/+* compared to *APP-PSEN1/+* mice (Fig. 5C). However, the number of microglial cells surrounding the Aß plaques (fig. S3A), the total microglial load (fig. S3B), or the morphology of microglia distal to Aß plaques (fig. S3C) was not altered in PHD3-deficient mice. To test whether phagocytosis of Aß could be altered by PHD3 deficiency, we measured the microglia uptake of Aß using in vivo labeling with methoxy-X04, a fluorescent compound that can cross the blood-brain barrier and has high binding affinity for Aß (*10*). Flow cytometry analysis showed a trend to increase the proportion of phagocytic microglia in *Egln3^−/−^*; *APP-PSEN1/+* compared with those in *APP-PSEN1/+* mice (Fig. 5D). Correspondingly, a decrease was observed in the total cortical levels of monomeric Aß_1-42_ and a trend in Aß_1-40_ (Fig. 5E) by enzyme-linked immunosorbent assay (ELISA). To study Aß plaque deposition, we stained cortical brain sections with thioflavin-S (Thio-S), which labels ß sheet structure–containing proteins like Aß, and with an anti-Aß antibody. We first estimated the percentage of the cortex covered (Thio-S or Aß load) by dense-core Aß plaques observing no differences between groups (Fig. 5F and fig. S3D). However, we observed a trend to increase the total number of Aß plaques in PHD3-deficient mice but with a reduced average Aß plaque size (Fig. 5F). Size distribution revealed that PHD3-deficient mice were characterized by an elevated abundance of small-sized Aß plaques with no changes in the bigger plaques (Fig. 5G). The uplift observed in Aß plaques burden could be attributed to core-dense and not filamentous Thio-S–positive plaques (Fig. 5H).

**Fig. 6.**
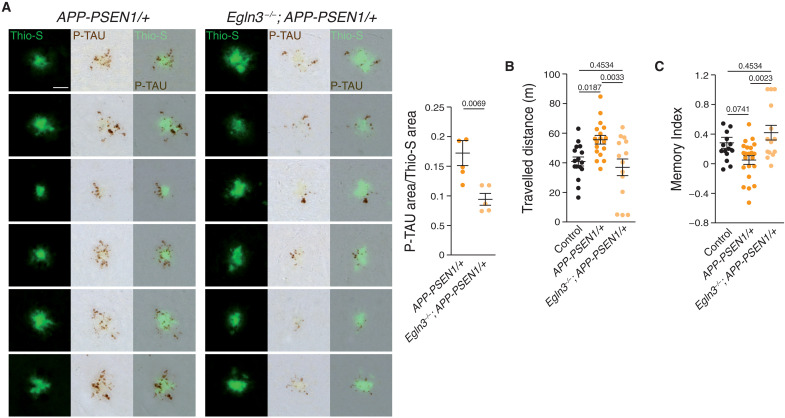
PHD3 deficiency decreases pathology and rescues behavioral alterations of an Aß mouse model. (**A**) Left: Aß plaques stained with Thio-S and anti–phospho-TAU (P-TAU). Right: Quantification of the ratio between P-TAU–immunoreactive area and Thio-S–positive area (24 plaques per mouse). Scale bar, 50 μm. *P* values from Student’s *t* test. (**B** and **C**) Spontaneous activity in the open-field test (B) and memory index in the novel object recognition test (C) were measured in 6-month-old mice. *P* values from ANOVA with Tukey’s posttest. All data are presented as means ± SEM. Data points depict independent 6-month-old control, *APP-PSEN1/+*, and *Egln3^−/−^*; *APP-PSEN1/+* mice used per experiment.

As microglial cells act as a physical barrier avoiding the spreading of the axonal pathology caused by Aß (*8*), we measured the number of dystrophic neurites found around Aß plaques using a phospho-TAU (P-TAU) antibody, a characteristic marker of this neuronal damage. A substantial decrease in P-TAU labeling was observed around the Aß plaques in the PHD3-mutant mice, indicating an enhanced shielding function of PHD3-deficient microglia (Fig. 6A).

Last, we investigated whether PHD3 deficiency could improve the cognitive impairment of AD mice. In an open-field test, *APP-PSEN1/+* mice exhibited hyperactivity when compared with WT and *Egln3^−/−^* control mice (Fig. 6B), as previously described in AD mouse models (*10*, *60*). In sharp contrast, PHD3 deficiency fully rescued this behavioral phenotype (Fig. 6B). To evaluate hippocampal-dependent memory, we used the novel object recognition test. *APP-PSEN1/+* mice showed reduced memory retention when compared with control mice, and the absence of PHD3 fully restored this memory deficit (Fig. 6C). Together, our results strongly indicate that PHD3 deficiency improves microglial responses to Aß, decreases local axonal pathology, and recovers behavioral deficits of an AD model.

### Microglial expression of PHD3 is sufficient to induce the IFNS and behavioral alterations

**Fig. 7.**
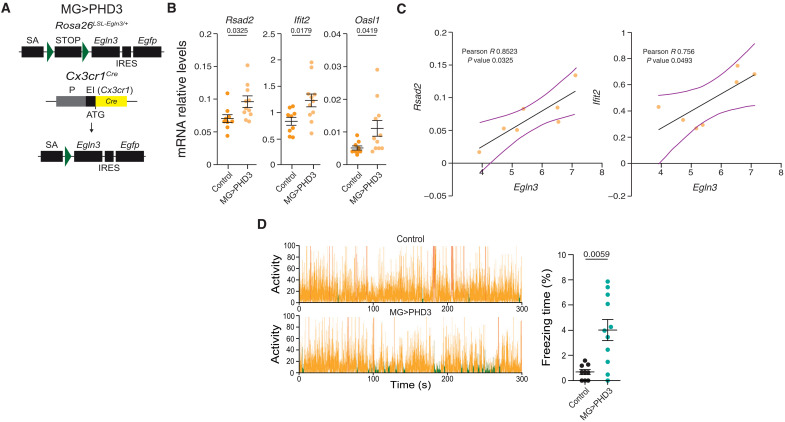
PHD3 overexpression is sufficient to induce the IFNS and behavioral alterations. (**A**) Schematic representation (non-scaled) of the *Cx3cr1^CRE/+^*; *Rosa26^LSL-Egln3/+^* (MG > PHD3) mouse model. SA, splicing acceptor. Green triangles indicate LoxP sites; STOP, Transcriptional STOP sequence; IRES, internal ribosome entry site; P, promoter; E1, exon 1. (**B**) qRT-PCR analysis of the expression of the IFNS in control and MG>PHD3. (**C**) Correlation between IFNS and Egln3 mRNA expression levels in isolated microglia from MG>PHD3 mice. (**D**) Control and MG>PHD3 mice were recorded in a chamber equipped with a high sensitivity weight detection system for 300 s. Orange, no freezing; and green, freezing. Right: Freezing time (%). All data are presented as means ± SEM. Individual points in the graphs indicate independent 9-month-old mice used per experiment. *P* values from two-tailed Student’s *t* test (B and D) and Pearson’s correlation test (C). *R*, Pearson’s correlation coefficient.

The IFNS is highly relevant for microglial function both during development (*46*) and in pathology (*20*, *45*). In both cases, the activation of the IFNS has been linked to signals from neurons that can be detected by microglia, which include double-stranded RNA (*46*) and mitochondrial DNA (*61*), and downstream pathways (*45*). We propose here a cell autonomous mechanism involved in the activation of the IFNS in microglia, by derepressing the IFNS through induction of the PHD3-FOXO3 interaction. To prove that the activation of PHD3 in microglia is sufficient to induce the IFNS in the absence of pathology, we developed a mouse model with overexpression of *Egln3* at the *Rosa26* locus in a CRE-dependent manner (fig. S4A). First, we validated the model by crossing *Rosa26^LSL-Egln3/+^* with a ubiquitous CRE::ERT2 line (*Ubc^Cre::Ert2/+^*), treating mice with tamoxifen, and estimating the degree of *Egln3* overexpression in kidney and brain (fig. S4B). We then crossed the *Rosa26^LSL-Egln3/+^* with the *Cx3cr1^Cre/+^* mouse line (Fig. 7A), which strongly recombines in microglia from development (*62*), and verified the up-regulation of *Egln3* in isolated microglia (*Cd11b* positive; fig. S4C). To investigate whether PHD3 expression in the absence of Aß pathology was sufficient to induce the activation of the IFNS, we measured the mRNA levels of several genes included in the IFNS in mRNA extracted from brain cortex, showing an up-regulation of the IFNS (Fig. 7B and fig. S4D). Isolated microglia showed a significant correlation between the *Egln3* and the IFNS mRNA expression levels (Fig. 7C and fig. S4E).

As microglia has an important role during development (*1*, *2*, *46*) and we have recently shown that activation of microglia during the perinatal period may associate with behavioral deficits (*63*), we measured the freezing time in freely moving mice. We found a clear increase in freezing time when compared to control mice (Fig. 7D), suggesting that PHD3 and its associated IFNS overexpression are sufficient to alter brain function.

## DISCUSSION

We have previously described HIF1 as a master regulator of AD microglial transcription (*23*). Here, we show that *Egln3*, encoding for PHD3, is a HIF1 target gene, whose expression is highly restricted to AßAM. In vitro, we observe that oAß-mediated induction of HIF1 and PHD3 expression correlates with the activation of the IFNS in microglia. We also show that FOXO3 is regulated by PHD3 and a repressor of the IFNS, as previously described in bone marrow macrophages (*44*) and demonstrated here in microglia. In vivo, PHD3 deficiency leads to a microglial transcriptional reprogramming, characterized by increased DAM and reduced IFNS, which results in a better AßAM fitness. PHD3 absence also improves the neuropathology and behavioral deficits of an AD mouse model. Last, we demonstrated that PHD3 expression in microglia is sufficient to induce the IFNS in the absence of Aß pathology and to alter brain function, strongly suggesting a cell autonomous mechanism.

We have previously shown that the HIF1-mediated transcriptional program is a characteristic of AßAM (*23*), mainly due to the low perfusion of Aß plaques (*24*). Although not demonstrated in vivo, the role of HIF1 in microglia had been inferred from that in monocyte-derived cells (*30*, *40*), where it has an important contribution in inducing a pro-inflammatory phenotype, characterized by a metabolic switch to glucose fermentation (lactic acid generation) and production of TNFα and IL-1ß (*28*, *29*). However, several research groups have challenged this presumed HIF1 role in microglia by showing that (i) resting microglia is more fermentative than the rest of brain cells (*64*) and activation of microglia in physiology (*65*) or pathology (*23*) associates with up-regulation of the mitochondrial electron transport chain encoding genes; (ii) hexokinase 2 is almost exclusively expressed in brain by microglia and up-regulated in DAM, and its deficiency associates with mitochondrial microglial dysfunction (*66*); and (iii) genetic overexpression of HIF1 (VHL conditional mutation) in microglia reduces mitochondrial gene expression, DAM, and proliferation (*23*). Our data further support differential consequences for HIF1 accumulation in microglia, with no associated TNFα and IL-1ß induction and increased IFNS. Hence, our findings unveil an unexpected role of the hypoxia signaling pathway regulating viral responses in microglia, similar to what was observed in other cell models (*67*). Two microglial populations express HIF1 in both human (*32*) and AD mouse models [(*23*) and this report], DAM and IFN microglia, but only the later expresses the IFNS. Although both HIF1 and FOXO3 were proposed as DAM drivers in human iPSC-derived microglia, simultaneous knockdown of HIF1 and FOXO3 (and also FOXP2) resulted in modestly reduced DAM but elevated IFNS expression (*32*), supporting a role for the PHD3-FOXO3 interaction downstream of HIF1 and suggesting that it may gate the transition between these two cellular states. Our data also point to trans-criptional regulation combined with niche factors [hypoxia and hypoperfusion; (*24*)] as contributors to sculpt the AßAM responses.

Although no previous studies have linked PHD3 with the activation of antimicrobial responses, PHD3 is elevated in macrophages from patients suffering chronic inflammatory diseases such as Crohn’s disease or ulcerative colitis (*33*). The absence of PHD3 has been associated with clinical improvement in mouse models of ulcerative colitis and acute lung injury, through HIF-independent mechanisms (*35*). However, worse outcomes have been observed in a sepsis mouse model due to an enhanced inflammatory response in a HIF1-dependent manner (*34*). 

We propose that the IFNS response mediated through the PHD3-FOXO3 pathway in AD microglia is maladaptive, inducing the activation of microglial cells against a non-real threat, such as a viral infection that is not taking place in the brain of AD mouse models. Similar “sterile” (in the absence of pathogen) activation of immune cells has been observed in the peripheral immune system and can be pathogenic (*68*). Intriguingly, Aß has recently been shown to have antimicrobial properties (*69*), polymorphisms in several IFNS genes were found associated to AD (*70*), other AD risk genes are proposed to activate the IFNS (*71*), and IFNS expression is up-regulated in AD patients carrying the *TREM2^p.R47H^* variant (*72*). The present findings indicate that modulation of the PHD3-FOXO3 pathway in microglia could offer a therapeutic target for AD.

## MATERIALS AND METHODS

### Mice

Mice were housed under controlled temperature (22°C) and humidity conditions in a 12-hour light/dark cycle with ad libitum access to food and water. Housing and treatments were performed according to the animal care guidelines of the European Community Council (86/60/EEC). All animal procedures were conformed under the Spanish law and approved with numbers 26/04/2016/064, 06/04/2020/050, and 17/10/2023/089 (“Consejería de agricultura, pesca y desarrollo rural. Dirección general de la producción agrícola y ganadera”). B6.Cg-Tg(APPswe,PSEN1Δ9E)85Dbo/J (*APP-PSEN1/+*; stock number 34832) (*73*), B6J.B6N(Cg)-Cx3cr1^tm1.1(cre)Jung^/J (*74*) (*Cx3cr1^Cre/+^*;, stock number 025524), B6.129-Hif1a^tm3Rsjo^/J (*Hif1a^Flox/Flox^*; stock number 007561) (*28*), and UB-CRE::ERT2 (B6.Cg-*Ndor1^Tg(Ubc-CRE::ERT2)1Ejb^/1 J*; stock number 007001) (*75*) mice were obtained from the Jackson Laboratory. *APP_751_SL/+* mice (Sanofi) (*76*) were provided by Transgenic Alliance-IFFA-Credo. *Egln3^−/−^* mice were a generous donation from P. J. Ratcliffe’s laboratory (*52*). WT mice were C57/Bl6. To activate CRE::ERT2-mediated recombination, mice were fed for 30 days with a tamoxifen diet (400 mg of tamoxifen citrate/kg; Envigo). All experiments were performed with balanced number of male and female mice. *Rosa26*-LoxP-stop-LoxP-*Egln3* mice were generated using a previously described CRISPR-Cas9 editing method (*77*). In the knock-in allele, *Egln3* coding region is linked to an internal ribosome entry site–green fluorescent protein (GFP) reporter element and placed downstream of the endogenous Rosa26 promoter connected to its transcript with splice acceptor elements. Conditional expression is achieved by insertion of a loxP-flanked transcriptional stop cassette between the Rosa26 promoter and *Egln3* coding region. *Egln3* murine cDNA (GenBank, AJ310548.1) was subcloned from pCMV-SPORT6 plasmid (Genomics Online, no. ABIN3831109) in the Bam HI and Xho I sites of pENTR 1A dual vector (Thermo Fisher Scientific, no. A10462) using the primers Bam HI–phd3-F and Xho I–phd3-R and then inserted by LR reaction in pR26-GFP-Dest plasmid (Addgene, no. 74283) using the Gateway LR Clonase system (Invitrogen, no. 11791-019). The resulting plasmid (PHD3-pR26-GFP-Dest) was used as the targeting vector for inserting *Egln3* cDNA into *Rosa26* locus. To achieve Cas9–mediated knock-in into the *Rosa26* locus, we used sgRosa26-1 RNA as previously described (*77*) from the plasmid pU6-sgRosa26-1_CBh-Cas9-T2A-BFP (Addgene, no. 64216). Targeting of the construct to the *Rosa26* locus was performed in FVB-fertilized zygotes via pronuclear co-injection with a mixture of Cas9 protein (50 ng/μl; PNA BIO Inc., no. CP01), sgRosa26-1 RNA (10 ng/μl) and PHD3-pR26-GFP-Dest targeting vector (10 ng/μl), in collaboration with the University of Seville animal facility CEA-Oscar Pintado. Founders were screened for cassette insertion with R26F2/SAR (*77*) and pR26-fwd/PHD3-rev primer combinations. Correct targeting into *Rosa26* locus was confirmed by PCR amplification of the external 5′ region with r26 fwd/r26rev primers (*78*). Cas9 off-target activity was assessed in the founder mice as described previously (*77*). Founders were crossed to WT FVB mice to generate F1 animals, and inheritance of the knock-in allele was confirmed by PCR amplification and by Sanger sequencing using r26fwd and r26rev primers. CRE::ERT2-mediated recombination was induced by tamoxifen (Sigma-Aldrich, no. T5648) injection in the UB>PHD3 mouse model and by feeding mice with a tamoxifen containing diet (Envigo) for 1 month in *Cx3cr1^CRE::ERT2/+^*; *Hif1a^Flox/Flox^* mice. For injection in the UB>PHD3 mouse model, tamoxifen was prepared in corn oil and intraperitoneally administered 2- to 2.5-month-old mice at a dose of 0.18 mg/g of body weight at a regimen of 1 injection per day for 3 to 5 consecutive days. Corn oil was intraperitoneally injected in the vehicle-only treated animals. Animals were euthanized 2 weeks after the first tamoxifen dose, and tissues were collected for RNA extraction.

### Flow cytometry

#### Microglia isolation from adult mouse brains

Mice were anesthetized and transcardially perfused with phosphate-buffered saline (PBS; Gibco) followed by Hanks’ balanced salt solution (-CaCl_2_/-MgCl_2_) (Gibco), and cortices were dissected and then dissociated using a Tissue Chopper (Vibratome, 800 series). Chemical digestion was performed with a mix of papain (Worthington) (8 U/ml) and deoxyribonuclease I (DNAse I; Sigma-Aldrich) (80 Kunitz U/ml) followed by a Percoll (GE Healthcare) gradient at 90% in PBS (v/v). Cells were stained with primary conjugated monoclonal antibodies CD11b–allophycocyanin (APC) and CD45–phycoerythrin (PE) (eBioscience, 1:200) at 4°C for 30 min. Staining with isotype control-APC and isotype control-PE (eBiosciences, 1:200) was used as a negative control. Both control and experimental samples were simultaneously incubated with anti-CD16/CD32 blocker antibody (eBioscience, 1:200). 7-Aminoactinomycin D (7-AAD) (BD Pharmingen) was added (1:100) to confirm alive microglial isolation as previously described (*23*). Cells were washed and sorted using FACSAria Fusion (Becton Dickinson) flow cytometer, and data were acquired and analyzed with FACSDiva software 8.0 (Becton Dickinson). The gating strategy and data analysis were performed according to guidelines and previous reports (*79*, *80*), with debris and dead cells discarded by forward and side scatter patterns (fig. S2A). Microglia subpopulation selection was performed on a contour density plot scaled to 15% probability, and a second microglial subpopulation was considered to be present when more than one independent focus was found with these settings. Percentages are relative to the total number of individual cells.

#### In vivo phagocytosis assay

We calculated the percentage of microglia from total single cells incorporating methoxy-X04 (Tocris Biosience) as previously described (*10*). Twelve-month-old mice were injected with methoxy-X04 (10 mg/kg) in 50% dimethyl sulfoxide (DMSO)/50% NaCl 0.9% intraperitoneally. After 3 hours, we isolated microglia using the described FACS protocol, and cells were analyzed in FACSCanto II (Becton Dickinson). Negative threshold for methoxy-X04 was established considering microglia from control mice (not Aß depositing) and *APP-PSEN1/+* mice without methoxy-X04 injections.

### Cell culture

#### HEK293T cell line cultures

Cells were grown in Dulbecco’s modified Eagle’s medium (Gibco) with 10% fetal bovine serum (Gibco) and 1% penicillin/streptomycin (Gibco) in a water-saturated atmosphere of 5% CO_2_ and 5% air. Cells were detached by trypsinization with 0.25% trypsin-EDTA (Gibco). Cells were always plated at 30 to 50% confluence to prevent anaerobic conditions.

#### Primary microglial cell cultures

Primary microglial cultures were prepared as previously described (*81*) from 1- to 5-day-old WT or *Egln3^−/−^* mouse brains.

#### In cellulo treatments

##### DMOG

Cells were incubated for 6 hours or 24 hours with 1 mM (HEK293T cells) or 0.1 mM (primary microglial cells) DMOG (Sigma-Aldrich) dissolved in DMSO (Sigma-Aldrich). A similar amount of DMSO was added to control cultures.

##### MG132

Cells were incubated for 4.5 hours or 6 hours with 12,5 μM (HEK293T cells) or 10 μM (primary microglial cell cultures) MG132 (Sigma-Aldrich) dissolved in DMSO (Sigma-Aldrich). A similar amount of DMSO was added to control cultures.

##### oAß treatment

We followed a published protocol (*47*) summarized as follows: We allowed synthetic lyophilized Aß_1-42_ peptide (human sequence; AnaSpec) to equilibrate at 20°C for 30 min before it was resuspended and diluted to 1 mM in 1,1′,1″,3,3′,3″-hexafluoro-2-propanol. After evaporation, peptide films were dried in a Speed Vacuum and stored at −80°C. Peptide films were resuspended to 5 mM in DMSO for 10 min. To form the oAß (*82*), we diluted the 5 mM DMSO solution to 100 μM in cold PBS, vortexed for 30 s, and incubated overnight at 4°C. Before use, the Aß-PBS solution was further diluted in culture medium to final concentration of 1 μm. Aß was tested as endotoxin free using the ToxinSensor Chromogenic LAL Endotoxin Assay Kit from GenScript.

##### Small interfering RNAs

Primary isolated microglial cell cultures were transfected with small interfering RNAs (siRNAs; 26.67 nM) for 48 hours, using Viromer Blue (Lipocalyx) as a transfection reagent following the manufacturer’s instructions. As a control, a nonspecific siRNA against *Drosophila melanogaster Sima* (*Hif1a* homolog) was used as in (*83*).

##### Transfections

HEK293T cells were transfected with different plasmids at different concentrations for 24 hours, using TurboFect (Thermo Fisher Scientific) as a transfection reagent following the manufacturer’s instructions.

Plasmids used: pCDNA3 (Thermo Fisher Scientific); pCDNA3-PHD1::V5 (*84*); pCMV6-PHD3::V5 (*84*); pCMV6-FOXO3::MYC-DDK (Origene); pCMV6-FOXO3(P426A/P437A)::MYC-DDK (this manuscript); pEF1 (Thermo Fisher Scientific); and pEF1-HIF1a::DDK.

### RNA extraction and RT-qPCR

#### Primary cultures, FACS-isolated microglia, and mouse brain and kidney samples

RNA was extracted using TRIzol reagent (Life Technologies). RNA samples were treated with PerfeCTa DNase I (Quanta Biosciences) and copied to cDNA using qScript cDNA SuperMix (Quanta Biosciences). cDNA from FACS-isolated microglia was amplified following the protocol described in the “Microarray” section. Real-time RT-qPCR was performed for all samples in a ViiA7 Real-Time PCR System (Applied Biosystems) using Power SYBER-Green PCR Master Mix or Taqman (Applied Biosystems). Oligonucleotides sequences are available under request.

### Protein extraction, immunoprecipitation, and Western blot

For primary microglia cell cultures and brain tissue, total proteins were extracted using TRIzol reagent (Life Technologies) according to the manufacturer’s instructions. An reducing agent and detergent compatible (RC-DC) protein assay kit (Bio-Rad) was used for quantifications. For HEK293T cells, total proteins were extracted using a lysis buffer [100 mM NaCl, 20 mM tris-HCl (pH 7.4), 5 mM MgCl_2_, 0.5% (v/v) Nonidet P-40, 1× proteases inhibitor without EDTA, and endonuclease, 1:2000]. The Pierce 660 Protein Assay Reagent (Thermo Fisher Scientific) was used for quantifications. For immunoprecipitation, anti-FLAG magnetic beads (Sigma-Aldrich) were used and incubated at 4°C overnight with the protein extracts. To elute proteins, we used 0.5 M ammonium hydroxide and 0.5 mM EDTA (pH 10). The eluate was neutralized by addition of 10% v/v 1 M tris (pH 7) before addition of Laemmli buffer. Western blots were performed using standard procedures. The antibodies used were anti-HIF1α (Cayman, 1:500), anti-FOXO3 (Cell Signaling Technology, 1:1,000), anti-V5 Tag (Cell Signaling Technology, 1:1,000), anti-FLAG horseradish peroxidase (HRP) conjugated (Sigma-Aldrich, 1:2,000), and anti–β-actin (Abcam, 1:5,000). HRP-conjugated anti-rabbit (1:10,000) or anti-mouse (1:10,000) antibodies and a Western ECL Substrate kit (Bio-Rad) were used for signal detection.

### In situ hybridization and immunostaining

#### Brain in situ hybridization

Brain tissues were cryoprotected in sucrose, embedded in OCT compound (Tissue-Tek) and kept at −80°C. Coronal slices (20 μm) were obtained with a cryostat (Leica). The RNAScope 2.5 [Advanced Cell Diagnostics (ACD)] protocol was used to detect *Egln3* mRNA (ACD) according to the manufacturer’s instructions, using a HybEZ oven (ACD). Subsequent immunostaining was performed for microglia staining (with the IBA1 marker) and nuclear staining [4′,6-diami-dino-2-phenylindole (DAPI) dye]. After the RNAScope 2.5 protocol, slices were incubated for 10 min in PBS/Triton X-100 0.3% (v/v) and washed in PBS. Samples were incubated with anti-IBA1 antibody (Wako, 1:500) overnight at 4°C. Slices were then incubated with Alexa Fluor 488 anti-rabbit (Invitrogen, 1:400) for 1 hour at room temperature and DAPI (Sigma-Aldrich, 1:1,000) stained before mounting with Fluoromont-G (Southern Biotech). Images were acquired using a confocal microscope (Leica SP2). Quantification of the number of *Egln3*^+^ ISH dots per microglia (IBA1 immunoreactive) was performed in confocal images in a 100-μm-diameter circumference with the center in the Aß plaques (Aß) and in microglia outside the circumference (non-Aß). Aß plaques were identified using their autofluorescence when excited with 408-nm light (*24*).

#### Brain immunostaining

The brain was removed from perfused mice with PBS and immediately fixed overnight (15 hours) at 4°C with 4% paraformaldehyde (PFA) in PBS. The brain was paraffin-embedded using an automatic tissue processor (ASP300S, Leica) and paraffin blocks cut in 20-μm-thick coronal sections using a microtome (RM2255, Leica). Immunostaining was performed according to standard protocols. Primary antibodies used: anti-IBA1 (1:500), anti-Aß 6e10 (1:500), and anti–P-TAU AT8 (1:500). For immunohistochemistry, Envision+ kit (DAKO) was used for chromatic staining. Secondary antibodies were added, and the reaction was developed with 3,3-diaminobenzidine (DAKO). For immunofluorescent studies, we used secondary antibodies anti-mouse or anti-rabbit conjugated with Alexa Fluor 488 or Alexa Fluor 568. Thio-S and DAPI staining were used as counterstains.

### Image quantification

#### Microglia density estimation

Unbiased stereological analysis was accomplished by systematic random sampling using a CAST Grid System (Olympus). A 25% of complete hemicortex was sampled with dissectors of 106,954.7 μm^2^ in three slices per mice from −1.7 to −2.06 mm relative to bregma. Cortical area was manually outlined at ×4 magnification. Microglia number in dissectors was quantified by IBA1 marker colocalization with nuclear DAPI staining at ×20 magnification. For each hemicortex, total sample area was obtained by multiplying dissector area by number of dissectors. Microglial density was calculated by dividing total microglia number by total sampled area using the Cavaleri method.

#### Microglia proximity index

Proximity index was calculated as the proportion of microglial cells in contact with each Aß plaque considering all microglia closer than 40 μm from Aß plaque’s border. We analyzed randomly selected compact plaques of similar size (15 plaques from three mice per genotype). Sampling area was the whole cortex from −1.94 to −2.3 mm relative to bregma. Images consisted in maximum intensity projections generated from *Z*-stacks using a New CST BX61 (Olympus) microscope at ×40 magnification. Aß plaques were delimited by using Fiji software, transforming pictures into 8-bit images, applying a lower threshold of 0 and an upper threshold of 255, and using analyse particle tool. The resulting particle was submitted to enlarge function to extend its periphery 40 μm. All microglia whose soma was within the extended halo or touching its periphery were quantified by IBA1 marker colocalization with nuclear DAPI staining. Separately, microglia directly in contact with the Aß plaque and within this halo were also quantified. The ratio between microglia directly in contact with Aß plaque and total microglia number in each extended halo constituted the microglial proximity index.

#### Microglial, Thio-S, and Aß load

Brain slices were stained with either IBA1, Thio-S, or an anti-Aß antibody. Measurements were performed in superimages, blind to the genotypes. Three cortex superimages per mouse were obtained at ×10 magnification from −1.7 to −2.3 mm relative to bregma with New CAST BX61 (Olympus) and using the same microscope setting for all the samples. Fiji software was used to analyse the superimages by transforming them into 8-bit, selecting the cortical area of interest manually, and subtracting areas with obvious noise or artifacts like folded tissue. We created a segmented binary mask by using the same lower and upper thresholds all along the samples for each staining. “Analyse particles” function was used to calculate the total area covered by all the particles contained in the segmented image. The load was defined as the percentage of occupancy by the particles divided the total cortical area studied and multiplied by 100. We also quantified the number of plaques per area (density) and the individual plaque’s area.

#### Microglia morphology

Individual microglial cells from the dorso-lateral cortex (12 cells from three slices per mouse) were randomly pictured using the same acquisition parameters. Images consisted in maximum intensity projections generated from Z-stacks in an Olympus BX61 microscope at ×100 magnification, from −1.70 and − 2.06 mm relative to bregma. Maximum intensity projections were obtained for further analysis. We counted the number of primary branches (emerging directly from the soma) and the number of intersections (Scholl index) with a circumference of 667.91 μm^2^. We also calculated the percentage of area covered by individual microglial cells in a circumference of 1108.24 μm^2^ with and without the soma (from circumference of 155.02 μm^2^).

#### Compact and filamentous Aß plaque density

All Thio-S–positive plaques bigger than 55 μm^2^ were quantified in the whole cortex and classified as filamentous, if no dense core was visible, or compact plaques, in which a solid and compact core was identifiable. Plaques were visualized at ×20 magnification in CAST Grid System (Olympus).

#### P-TAU load

Plaques were randomly selected by Thio-S staining, and the Thio-S and P-TAU area were estimated using Fiji in individual plaques from maximum intensity projections generated from *Z*-stacks imaging in an Olympus BX61 microscope at ×40 magnification. Results are presented as a percentage of P-TAU area per amyloid plaque area.

### Microarray

RNA quality was analyzed using an Agilent 2100 Bioanalyzer (Agilent). Only samples with RNA integrity number higher than 7 were used. The total RNA extracted from FACS-isolated microglia was amplified and labeled using the GeneChip WT Pico Reagent Kit (Thermo Fisher Scientific). The amplified cDNA was quantified, fragmented, and labeled for hybridization to GeneChip Mouse Transcriptome 1.0 Array (Thermo Fisher Scientific) using 5.5 μg of single-stranded cDNA product and following protocols outlined in the user manual. Washing, staining (GeneChip Fluidics Station 450, Thermo Fisher Scientific), and scanning (GeneChip Scanner 3000, Thermo Fisher Scientific) were performed following the manufacturer’s guidelines. Raw data from the Expression Console extraction software (Thermo Fisher Scientific) were imported into the statistical program R (RStudio Inc.) using the LIMMA/Bioconductor package (*85*). The quality of the data was assessed using Array Quality Metrics package (*86*). Data were normalized using the robust multi-array method, and differential expression analysis was performed using the LIMMA/Bioconductor package (*87*). Gene expression data from *APP-PSEN1/+*, *5xfAD*, *APP_751_SL/+*, and *Egln3^−/−^* mouse models were analyzed by GSEA using biological processes C5-v5.2, KEEG, and the custom DAM, IFNS, and FOXO3_BS (table S2) (*44*).

### Human snRNA-seq data

#### Dataset

The snRNA-seq data used in this study were sourced from the publicly available dataset hosted at https://compbio.mit.edu/microglia_states/ from (*32*). The dataset, comprising both the metadata and gene expression count matrix, was downloaded in RDS format. To facilitate downstream analysis in Python, the RDS files were converted into comma-separated value (CSV) format for metadata and Matrix Market exchange (MTX) format for the count matrix, enabling compatibility with standard Python-based computational tools for single-cell data analysis.

#### Data preprocessing

Quality control metrics were applied to remove outlier cells from the dataset. Cells were filtered on the basis of the total number of counts, retaining those with at least 600 and no more than 9000 counts. Additionally, cells were required to express a minimum of 500 genes and a maximum of 3000 genes. To exclude cells with excessive mitochondrial content, a mitochondrial fraction cutoff of 0.1% was applied. The initial dataset contained 174,420 cells, and, following quality control, 168,373 cells remained. Raw count data were normalized using the “normalize_per_cell()” and “log1p()” functions from the Scanpy library (*88*) to standardize gene expression levels for subsequent analyses.

#### Clustering analysis

To replicate the cluster definitions from the original study (*32*), we used the Seurat clusters provided in the metadata, as the original cluster labels were not available. These Seurat-defined clusters were correlated with the highly expressed gene markers for each microglial state, as reported in the supplementary table of the original article. While most clusters could be mapped to the original labels, some were challenging in terms of clear correlation. For clusters that could not be confidently matched, we classified them as non-defined. Consequently, we defined specific microglial states, including microglia (MG)0, MG2, MG3, MG4, MG5/barrier-associated macrophages, MG6, MG7, MG8, MG10, MG11, MG12, and T cells. Uniform Manifold Approximation and Projection (UMAP) plot illustrating 11 distinct microglial states as identified by (*32*) in different colors. Each dot corresponds to an individual cell, sampled from six different brain regions: prefrontal cortex, hippocampus, mid-temporal cortex, angular gyrus, entorhinal cortex, and thalamus. Cells are clustered on the basis of their transcriptional profiles.

##### IFNS and FOXO3 BS signatures analysis

A predefined list of IFN and FOXO3 gene signatures from mice was applied to assess the correlation between these signatures and the microglial states in our single-cell dataset showing the expression of the IFN and FOXO3 BS gene signatures across various microglial states from the entorhinal cortex and hippocampus. For each cell, we calculated an expression score for both the IFN and FOXO3_BS signatures on the basis of the mean expression level of all genes associated with each signature. These calculated values, referred to as IFNS and FOXO3-BS, were then analyzed using the “rank_genes_groups()” function from Scanpy (*88*), which applies the Wilcoxon rank sum test. This approach allowed us to determine whether any of the signatures could serve as markers for any of the microglial states.

### Behavioral analysis

#### Locomotion activity

Mice were filmed in an open-field arena of 45 cm by 45 cm using an automatic tracking software (SMART). Mice were recorded for 15 min following the protocol from the international mouse phenotyping resource of standardized screens (IMPRESS; www.mousephenotype.org/impress/ProcedureInfo?procID=81). Traveled distance was calculated using the SMART software 3.0 (Panlab-Harvard Apparatus).

#### Novel object recognition test

Short-term memory was evaluated following a previously published protocol (*89*). Two objects were presented to each mouse for 15 min in a 45 cm–by–45 cm arena. After 1 hour, the mouse was exposed to one of the previous objects being the other replaced by a different one. A short memory index was calculated by subtracting the number of approaches to the “novel” object minus the number of approaches to the old object and divided by the total number of approaches. Recording and analyzing software was SMART 3.0.

#### Freezing behavior

The duration of freezing was estimated using a fully automatic startle and fear conditioning system (Panlab). Mice were introduced in a chamber equipped with a high sensitivity weight transducer system, and the immobility was measured using the PACWINCSFR software (Panlab), with a lower bound threshold of 18% and a minimal duration of 1 s.

### Aß ELISA

Frozen hemicortices were homogenized with a Dounce grinder (Sigma-Aldrich) in 4× w/v solution of PBS containing protease inhibitor cocktail (Roche, 1:25) and phosphatase inhibitor (Roche, 1:100). Samples were centrifugated at 600*g* for 5 min to pellet the nuclear fraction. Next, the supernatants were ultracentrifuged at 100,000*g* for 1 hour at 4°C to isolate PBS-insoluble proteins. This insoluble fraction was resuspended in 8.2 M guanidine-HCl, 50 mM tris-HCl, diluted to 5 M guanidine-HCl, and incubated at room temperature for 4 hours with occasional inversion to promote dissolution. Total protein concentration was quantified with RC-DC kit (Bio-Rad) and Aß_1-40_ and Aß_1-42_ concentrations were tested using human Aß_40_ and Aß_42_ ELISA kits (Invitrogen).

### Statistical analysis

All individual measurements constitute independent biological replicates. Samples with *n* ≥ 9 were evaluated for normal distribution using D’Agostino and Pearson’s omnibus normality test, and nonparametric tests were used if normality criterion was not achieved. Samples with *n* < 9 were analyzed using parametric tests. Comparisons between two groups were performed with two-tailed unpaired Student’s *t* test, whereas comparisons between more than two groups were done with analysis of variance (ANOVA) with Tukey’s test. Data are expressed as means ± SEM. *P* ≤ 0.05 was considered statistically significant. Statistical analyses and graphs were performed in GaphPad Prism (GraphPad).
