# Supplementary material for: Inactivation of the PHD3-FOXO3 axis blunts the type I interferon response in microglia and ameliorates Alzheimer’s disease progression
Source: Sci Adv. 2025 May 28;11(22):eadu2244. doi: 10.1126/sciadv.adu2244 (PMC12118632; doi:10.1126/sciadv.adu2244)
Supplement: Supplementary file 1 — Figs. S1 to S4 Legends for tables S1 to S7 [file sciadv.adu2244_sm.pdf]

Science Advances
AAAS

Supplementary Materials for

Inactivation of the PHD3-FOXO3 axis blunts the type I interferon response in microglia and ameliorates Alzheimer's disease progression

Manuel A. Sanchez-Garcia *et al.*

Corresponding author: Alicia E. Rosales-Nieves, aerosales-ibis@us.es; Alberto Pascual, apascual-ibis@us.es

Sci. Adv. **11**, eadu2244 (2025)
DOI: 10.1126/sciadv.adu2244

The PDF file includes:

Figs. S1 to S4
Legends for tables S1 to S7

Other Supplementary Material for this manuscript includes the following:

Tables S1 to S7

Fig. S1.

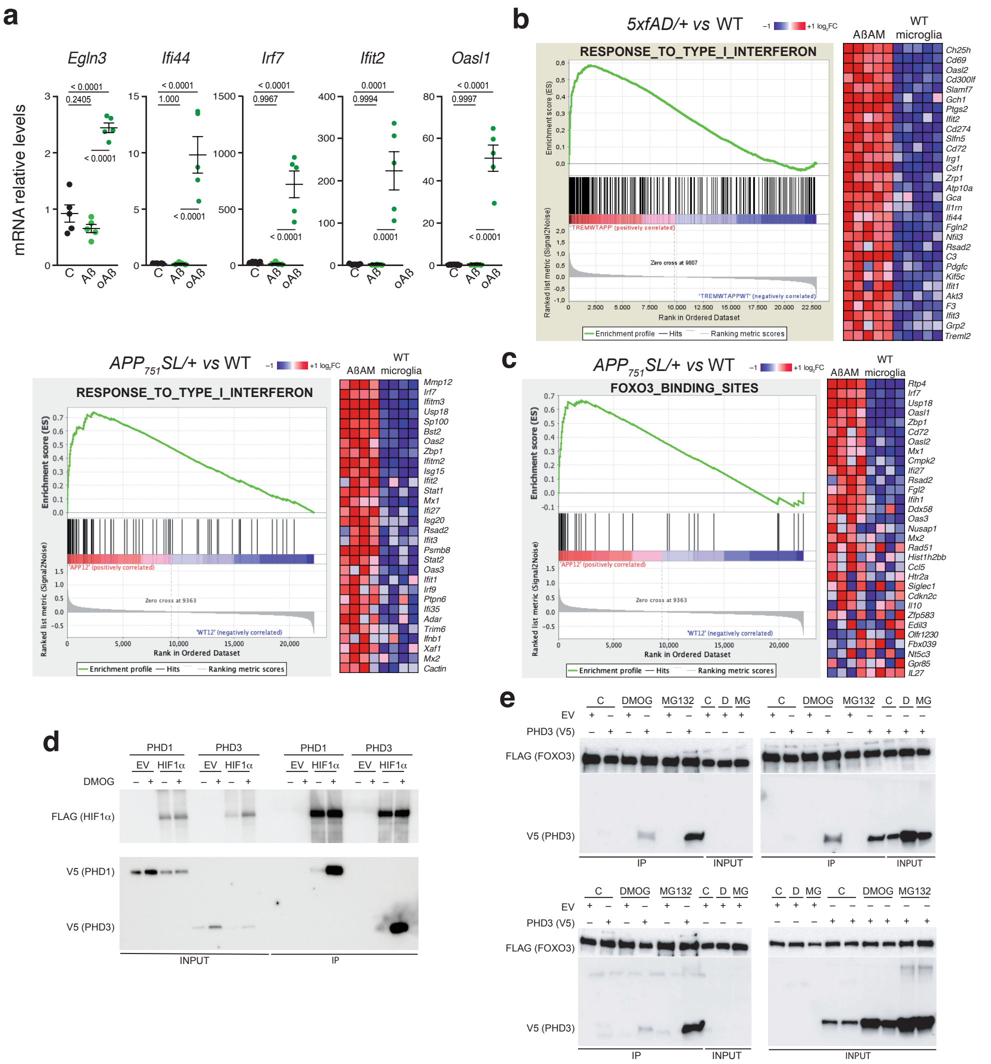

The AßAM from mouse models express the IFNS. **a**, mRNA levels of *Egln3* and IFNS were estimated in primary neonatal microglial cell cultures exposed to control (–), monomeric Aß (Aß), or oligomeric Aß (oAß) for 24 h. *p*-values from ANOVA with Tukey's post-test. **b**,**c**, Gene set enrichment analysis (GSEA) in adult microglia isolated from *5xfAD/+*, *APP-PSEN1/+*, and *APP₇₅₁SL/+* (**b** and **c**) Aß-depositing mouse models. Left panels, enrichment plots; right panels show the heatmap of the top 30 ranking leading edge genes. **d**, HEK-293T cells transfected with both PHD1-V5 or PHD3-V5 and an empty (EV) or HIF1α-FLAG (HIF1α) vector; and incubated with or without DMOG for 24 h. Upper panels show western blot with anti-FLAG (HIF1α) and bottom panels with anti-V5 (PHD1 and PHD3) with proteins extract (INPUT) and after immunoprecipitation (IP) with an anti-FLAG antibody. **e**, HEK-293T cells transfected with PHD3-V5 or an empty (EV), and a FOXO3-FLAG vector, and incubated with vehicle (C), DMOG (D) or MG132 (MG) for 24 h. Western blots with anti-FLAG (FOXO3) and anti-V5 (PHD3) in proteins extracts (INPUT) and after immunoprecipitation (IP) with an anti-FLAG antibody.

Fig. S2.

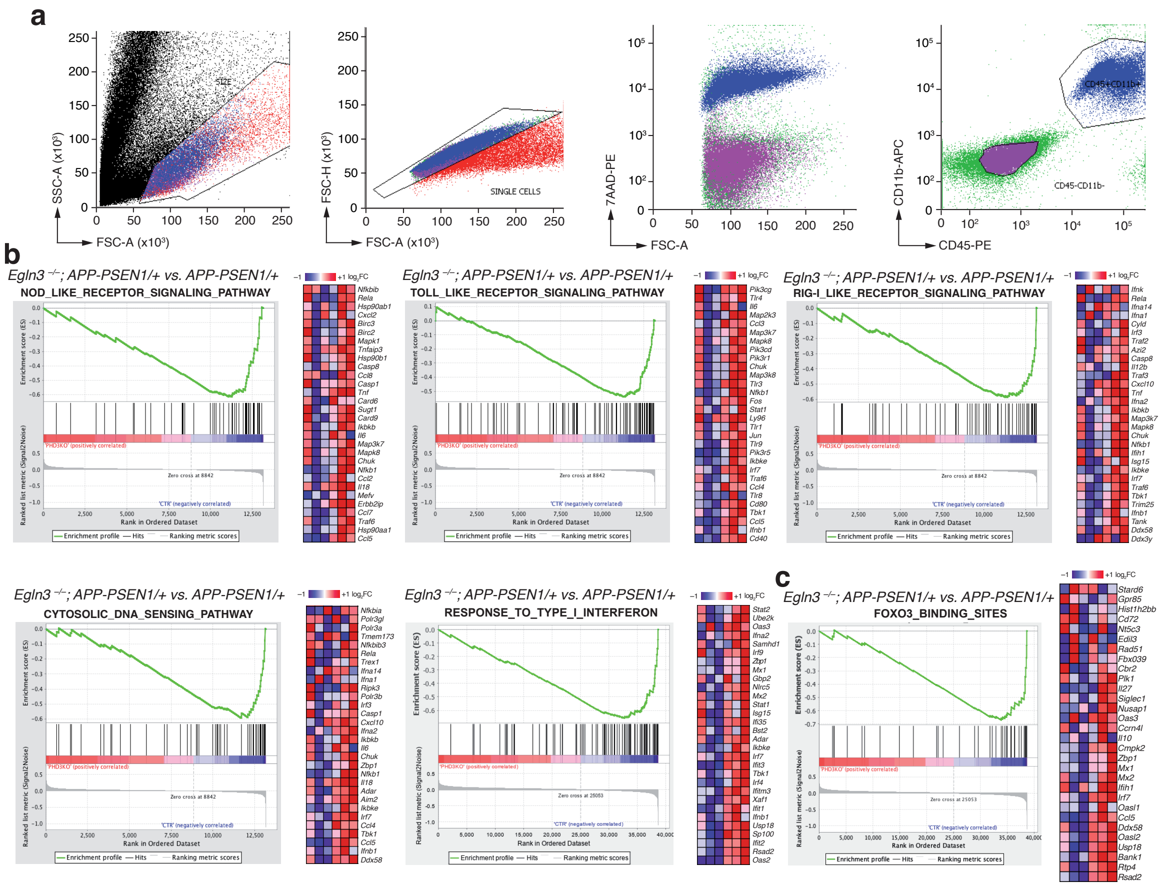

PHD3 deficiency abolishes the anti-viral transcriptional response of AßAM. a, Debris and dead cells were discarded by forward (FSC) and side (SSC) scatters dispersion of events (far left panel). Singlets of events were selected according to FSC height (FSC-H) versus area (FSC-A; left panel). 7-AAD staining was performed as control to confirm alive microglial cells isolation (right panel). Microglial cells, positive for CD45 and CD11b markers, were selected. **b,c**, GSEA of adult microglia from 12-month-old *Egln3*$^{-/-}$; *APP-PSEN1/+ versus APP-PSEN1/+* (left panels, enrichment plots). Right panels show the heatmap of the top-ranking leading-edge genes. FC: fold change.

Fig. S3.

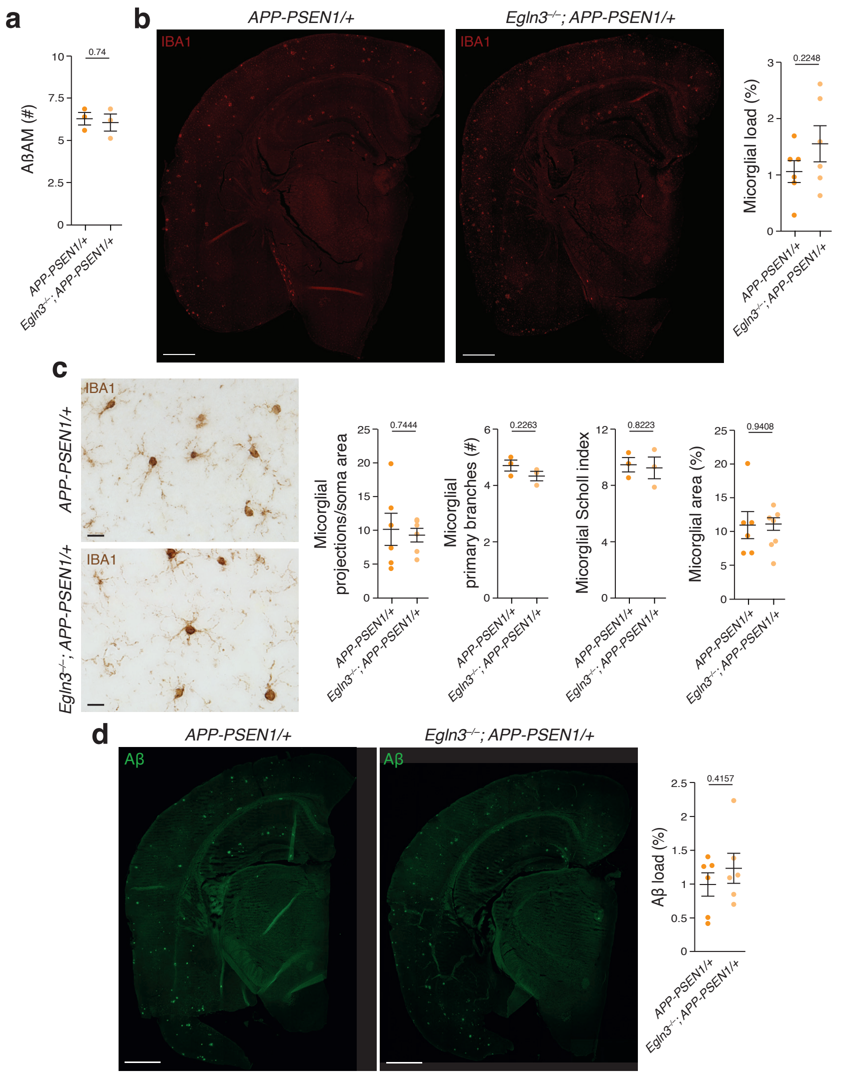

Histological characterization of the PHD3 deficient mouse model. **a**, Number (#) of microglial cells surrounding Aß plaques in *APP-PSEN1/+* and *Egln3*$^{-/-}$; *APP-PSEN1/+* mice. **b**, Representative cortical coronal sections from *APP-PSEN1/+* and *Egln3*$^{-/-}$; *APP-PSEN1/+* mice stained with IBA1. Scale bars: 500 µm. Right, microglial (IBA1) load. **c**, Morphologic characterization of non-plaque associated microglia from *APP-PSEN1/+* and *Egln3*$^{-/-}$; *APP-PSEN1/+* mice and stained with IBA1. Scale bars: 10 µm. Right graphs, microglial morphologic parameters. **d**, Representative cortical coronal sections from *APP-PSEN1/+* and *Egln3*$^{-/-}$; *APP-PSEN1/+* mice immunostained with an anti-Aß antibody. Scale bars: 500 µm. Right, Aß load. All data are presented as means ± s.e.m. Individual points in the graphs indicate independent 6-month-old mice used per experiment. *p*-values from two-tailed Student's *t*-test.

Fig. S4.

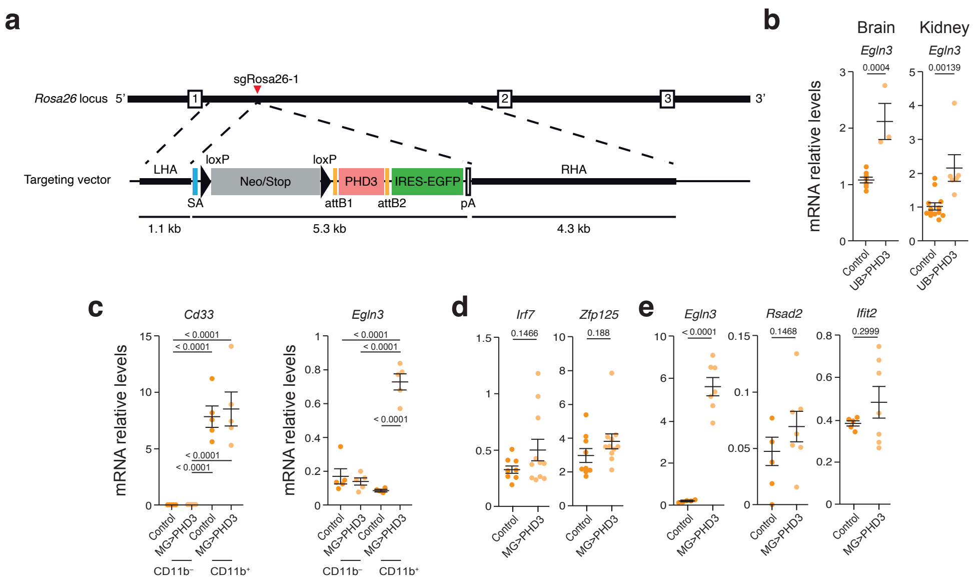

Characterization of the *Rosa26*$^{LSL\text{-}Egln3/+}$ mouse model. **a**, The scheme illustrates the strategy for insertion of the targeting cassette into the mouse *Rosa26* locus. Numbers in boxes indicate exons 1-3 of *Rosa26* locus. sgRosa26-1 single guide RNA directs Cas9 to the target site (red arrow) between the 1 kb and 4 kb fragments used as left and right homology arms in the targeting vector (LHA, left homology arm; RHA right homology arm). The following features are also displayed in the targeting vector: SA, splice acceptor site; Lox-P sites flanking the transcriptional Stop cassette (Neo/Stop); attB1 and attB2, site-specific recombination sites produced after Gateway LR reaction; PHD3, PHD3 cDNA cassette; IRES-EGFP, internal ribosomal entry site (IRES) followed by an EGFP reporter element; pA, polyadenylation site. Generation of the Cas9-directed double-strand break promotes the insertion of the cassette into the genome. In the targeted allele, PHD3 and EGFP transcripts are expressed from the *Rosa26* promoter through the SA site, **b**, qRT-PCR analysis of *Egln3* mRNA expression in *Ub*$^{CRE::ERT2/+}$; *Rosa26*$^{LSL\text{-}Egln3/+}$ (UB>PHD3) and control mice from brain (left panel) and kidney (right panel) samples. *Rps14* was used as a housekeeping gene. Results from the following mice were pooled together and assigned to a control group (control) as no differences were detected among them: *Ub*$^{CRE::ERT2/+}$; *Rosa26*$^{+/+}$ tamoxifen-treated mice; *Ub*$^{+/+}$; *Rosa26* $^{LSL\text{-}Egln3/+}$ tamoxifen-treated mice; *Ub*$^{+/+}$; *Rosa26* $^{LSL\text{-}Egln3/+}$ mock-treated mice, and *Ub*$^{CRE::ERT2/+}$; *Rosa26*$^{LSL\text{-}Egln3/+}$ mock-treated mice. **c**, qRT-PCR analysis of the expression of the *Cd33*

(microglial marker) and *Egln3* mRNA levels in microglia ($CD11b^+$) and other cells ($CD11b^−$) isolated from control and MG>PHD3 mouse model. **d**,**e**, qRT-PCR analysis of the expression of the IFNS (**d**,**e**) and *Egln3* (**e**) in bulk brain (**d**) and isolated microglia (**e**) from control and MG>PHD3 mice.

All data are presented as means ± s.e.m. Individual points in the graphs indicate independent mice used per experiment. *p*-values from two-tailed Student's *t*-test.

Table S1. (separate file)
Gene sets enriched in *5xfAD/+ versus* Wild-type microglia

Table S2. (separate file)
Gene Sets for DAM, IFNS and FOXO3 Binding sites (BS)

Table S3. (separate file)
Gene sets enriched in *APP-PSEN1/+ versus* Wild-type microglia

Table S4. (separate file)
Differentially expressed (DE) genes between *APP-PSEN1/+; PHD3* $^{-/-}$ *versus APP-PSEN1/+* microglia

Table S5. (separate file)
Gene sets enriched in *Egln3*$^{-/-}$*; APP-PSEN1/+ versus APP-PSEN1/+* microglia

Table S6. (separate file)
Gene sets downrepresented in *Egln3*$^{-/-}$*; APP-PSEN1/+ versus APP-PSEN1/+* microglia

Table S7. (separate file)
Gene sets downrepresented in *Egln3*$^{-/-}$*; APP-PSEN1/+ versus APP-PSEN1/+* microglia
